# Gradual edge enhancement in spiral phase contrast imaging with fractional vortex filters

**DOI:** 10.1038/srep15826

**Published:** 2015-10-29

**Authors:** Jikang Wang, Wuhong Zhang, Qianqian Qi, Shasha Zheng, Lixiang Chen

**Affiliations:** 1 Department of Physics and Laboratory of Nanoscale Condensed Matter Physics, Xiamen University, Xiamen 361005, China

## Abstract

In the spiral phase contrast imaging, the integer spiral phase plate (SPP) are generally employed to perform the radial Hilbert transform on the object. Here we introduce fractional SPP filters, instead of the integer ones, to investigate the gradual formation of edge enhancement for pure phase objects. Two spatial light modulators are used in our experimental configuration. One is addressed to display the pure phase object of a five-pointed star, while the other serves as a dynamic filter of fractional topological charge *Q*. Of interest is the observation of the complete reversal of the edge and background brightness by gradually changing the fractional vortices from *Q* = 0 to 1. The experimental results were well interpreted based on the OAM spectra of fractional SPP, which indicates that the filtered output image can be considered as a coherent superposition of all possible images that are individually resulted from the integer OAM filtering. Besides, we show that the spiral phase contrast effect can still be observed in real time for a rotating three-leaf clover. Our results may find potential applications in the optical microscopic imaging.

Spiral phase contrast imaging is based on the spiral phase filtering which is sensitive to the phase/intensity gradients of an object. This technique has been demonstrated a useful tool for image processing^1^, edge enhancement in microscopy^2^ and detection of an astronomical object^3^. The most commonly used filters in the spiral phase contrast imaging is the spiral phase plate with the topological charge 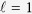. Owing to the symmetry of an integer SPP, in principle, all phase edges of a sample object are enhanced isotropically, independent of their local orientation. Furhapter *et al.* demonstrated that the resolution for phase jumps of a pure phase object is enhanced by orders of magnitude with the spiral phase method^4^. Bernet *et al.* obtained a quantitative reconstruction of both amplitude and phase information of an epithelial cheek cell sample by post-processing of a sequence of spiral-filtered images recorded with different rotational orientations of SPP^5^. Guo *et al.* demonstrated that the Laguerre-Gaussian spatial filter shows some advantages in achieving a high contrast edge enhancement with high resolution compared with the conventional SPP^6^. Yuan *et al.* showed that a Bessel-like amplitude modulated spiral phase filter can be further reduced the imaging diffraction noise compared with the Laguerre-Gaussian spatial filter^7^. Recently, Lauterbach *et al.* demonstrated an optical design that allows straightforward implementation of a phase contrast channel into a Stimulated Emission Depletion microscope in wide field and scanning modes^8^.

In contrast with the ideal SPP of 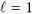, it has been shown that a slight modification of the phase structure of SPP can lead to the interesting shadow effect with a relief-like pattern^9^. By introducing the sine function or shifted sine function in conventional vortex phase distribution, Sharma *et al.* showed the edge can be selectively enhanced in any desired direction^10^,^11^. However, there are only a little attention paid to the fractional SPP with non-integer topological charge^12^. Davis *et al.* demonstrated that by rotating the fractional radial Hilbert mask where *Q* = 0.5, the orientation of the shadowing can be changed^1^. Situ *et al.* demonstrated both in theory and experiment that the directional edge enhancement can be obtained by utilizing a fractional or shifted vortex filter^13^. They also tried to observe the effect of fractional SPP filters on the degree of the edge enhancement^14^. But the results about the gradual formation of edge enhancement were not evident, as the complex character samples or biological specimen were used in their experiment. In contrast, we use a computer-controlled spatial light modulator (SLM) to display a simple pure phase object of five-pointed star, and use another SLM to serve as the dynamic fractional SPP with tunable topological charges. Thus we are able to observe that the brightness of the edge and background is reversed completely, by changing *Q* from 0 to 1, and therefore, resulting in an evident effect of gradual edge enhancement. Besides, we show that the spiral phase contrast effect can be still observed in real time for a rotating three-leaf clover with a fixed 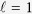. Based on the OAM spectrum of fractional SPP filters, our theoretical analysis also provides a deeper understanding to our observed effect.

## Results

### Theoretical analysis

The helical phase structure produced by SPP, exp(*iQφ*), is generally related to the orbital angular momentum (OAM) content of a light beam. It has been recognized that if the topological charge is an integer, for example, 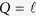, then each photon within the light beam carries a well-defined OAM of 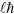
15. In a common experiment of spiral phase contrast imaging, the ideal SPP with 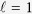 is placed in the Fourier plane of a 4f spatial filtering system. We can write the SPP filter function in the polar coordinates (*ρ*, *φ*) of the Fourier plane as, 
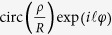
, where 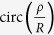 describes the circular aperture of radius R. The role that the SPP plays can be considered as a radially symmetric two-dimensional (2D) generalization of the 1D Hilbert transformation, since each straight line passing through the center of the SPP is equivalent to a 1D Hilbert transform filter^7^. We assume that the input object is mathematically described by a function in the real space, *g*(*r*, *ϕ*), which is placed at the front focal plane of the first lens. Then we obtain the Fourier transform of the object, *F*[*g*(*r*, *ϕ*)], at its rear plane. After passing the SPP, we can write the total field by multiplying the Fourier spectrum by the SPP filter function, namely,


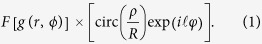


This modulated field of Eq. (1) is again Fourier transformed to yield a modified output image in the rear focal plane of the second lens, which reads,


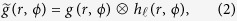


where ⊗ denotes the convolution operation, and 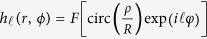, represents the Fourier transform of the SPP filter with an integer topological charge 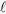. From the mathematical point of view, here 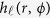 just plays the role of the point spread function of the system, which reads^16^,^17^,


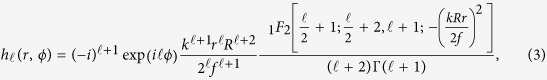


where *k* is the wave number, *f* is the focal length, _1_*F*_2_(·) is Hypergeometric function, and Γ(·) is the gamma function. In our scheme, we employ the fractional SPP of non-integer *Q*, instead of the integer ones, as the spatial filters, and aim to observe the gradual edge enhancement by varying *Q*. As a result, the modulated field previously described by Eq. (1) should be rewritten as,


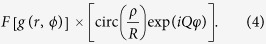


Recent years have also witnessed a rapidly growing interest in the fractional vortex states, owing to their high-dimensional quantum property^18^,^19^. As the integer OAM eigenstates form a complete, orthogonal and infinite basis, in principle, a fractional vortex can be expressed in terms of the OAM eigenstates as follows^20^,


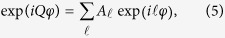


where 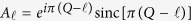 characterizes the weight of each OAM component. In this scenario, the output image, 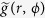, should take the following form,


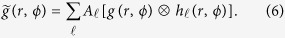


Equation (6) gives us an intuitive understanding of the role that a fractional SPP filter plays in comparison with an integer SPP. Specifically, the output image can be effectively considered as a coherent superposition of all possible images that are individually resulted from the SPP filtering with integer 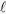, as is also shown below.

### Gradual formation of edge enhancement

**Figure 1:**
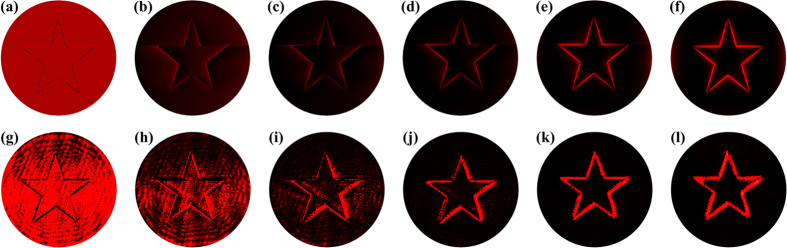
The simulation and experimental results. (**a**–**f**) The simulation results that are performed based on Eq. (6) while (**g**–**l**) The experimental observations that are recorded by the color CCD camera. (**a**,**g**): *Q* = 0, (**b**,**h**): 0.2, (**c**,**i**): 0.4, (**d**,**j**): 0.6, (**e**,**k**): 0.8, and (**f**,**l**): 1 (see video 1).

**Figure 2:**
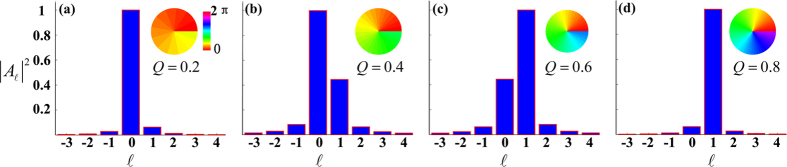
The OAM spectra for fractional SPP filters: (a) *Q* = 0.2, (b) 0.4, (c) 0.6, and (d) 0.8.

For comparison, we present both the numerical simulations and experimental observations in Fig. 1. The simulations are performed based on Eq. (6). The observations are recorded with the color CCD camera in the optical setup(see the Method section for details), where the topological charge are tuned from *Q* = 0 to 1 at an interval of Δ*Q* = 0.05. One can see clearly the good agreement between them, and an evident effect of the gradual edge enhancement. If *Q* = 0, as mentioned above, we just reproduce the input object. Generally, a pure phase object cannot be imaged or recorded, as the CCD camera is only an intensity-sensitive device. However, a delicate consideration to make the phase jump between the inside and outside area be strictly *π* enables a destructive interference occurs along the contour line of the star, and therefore, leading to the observation of the completely dark outline in a very bright background, see both Fig. 1(a,g). While Fig. 1(f,l) are those results in a traditional experiment of spiral phase contrast with an ideal SPP of 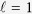, mathematically corresponding to Eq. (2). The key difference between Fig. 1(a,f), or equivalently Fig. 1(g,l), is the complete reversal of the edge brightness, namely, a sharp transition from the dark edge with a very bright background in Fig. 1(a,g) to the bright edge with an almost dark background in Fig. 1(f,l). Along this line, it is natural for us to expect that the gradual formation of edge enhancement, as the intermediate evolution between these two extremes, could be observed by simply changing *Q* from 0 to 1. As are shown by Fig. 1(b–e,h–k), both the simulation and experimental results confirm well our prediction. As *Q* is increasing, we observe that the contour line of the five-pointed star is turning bright while the background is becoming dark. This effect can also be interpreted well on the basis of Eq. (5) and (6). By plotting the OAM spectra of the fractional SPP in Fig. 2, we are able to give a more intuitive picture as to how the edge enhancement form gradually. As the topological charge is put in the range 0 < *Q* < 1, so the OAM components of 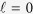 and 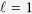 dominate in the OAM spectrum. According to Eq. (6), the output image can be approximately treated as a superposition of Fig. 1(a,f), which are weighted by the coefficients *A*_0_ and *A*_1_, respectively. As can be seen from Fig. 2, as *Q* is increasing, the weight *A*_0_ decreases such that the background resulted from Fig. 1(a) becomes darker. In contrast, *A*_1_ increases such that the edge brightness resulted from Fig. 1(f) becomes higher. Besides, the orientation-selective edge enhancement can also be observed in our case. As the fractional SPP we use here has a horizontal discontinuity along the x-axis, see the inset (c) of Fig. 1, so we can see that the edge enhancement of the horizontal sides of the five-pointed star is a bit suppressed, see Fig. 1(b,c,h,i) for example. This is consistent with that *Q* < 0.5 leads to a low-contrast edge^13^.

### Rotation of a clover with spiral phase contrast

**Figure 3:**
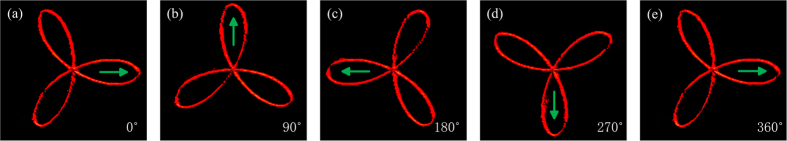
Experimental observations for a rotating three-leaf clover in a cycle. The green arrows indicate the orientation of the same leaf, and the rotation angles for each frame are indicated in the right-bottom corners (see video 2).

**Figure 4:**
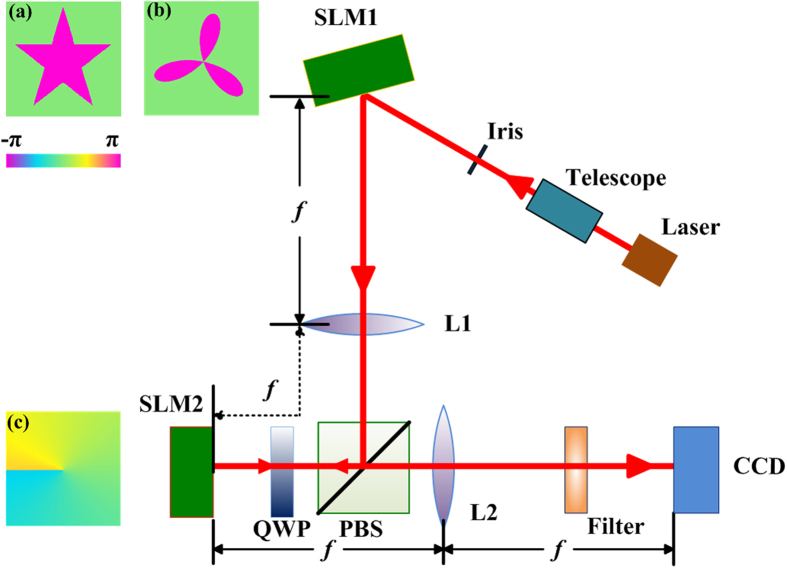
Schematic overview of the experimental setup, see the tex for details.

Besides, we consider that the SLM can act as a reconfigurable diffractive hologram, and allows an interactive manipulation with a response time comparable to the video displays. This provide us a convenient way to observe the realtime video of an object, such as, a rotating three-leaf clover. The phase profile of the three-leaf clover is also shown by the inset (b) of Fig. 4. Without loss of generality, we use the ideal SPP filter with 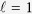 and the “perfect” edge enhancement is clearly observed. The setup is almost the same as Fig. 1, except for that the clover is set to be rotating in a constant angular speed of Ω = 0.13 rad/s. The experimental observation of some typical frames are shown in Fig. 3. The same leaf of the clover is marked by the green arrow, which indicates an anti-clockwise rotation. A subtle change of the brightness during the rotation, even for the same leaf, can be found. Actually, this brightness fluctuation is dependent on the specific spatial location where the leaf sweeps, see the accompanying video 2 for more details. We attribute this effect to the very slight misalignment of the SPP filter positioned in the Fourier plane.

## Discussion

In summary, we have presented a 4f optical system incorporating two SLMs to observe the spiral phase contrast image resulted from spatial filtering of fractional SPP. By carefully designing a pure object of five-pointed star with a phase jump along the contour, we have observed the gradual formation of edge enhancement by changing the fractional vortices from *Q* = 0 to 1, where, interestingly enough, the edge and background brightness can be completely reversed. The experimental results were well confirmed by our numerical simulations, and also interpreted visually based on the OAM spectra of fractional SPP filters, which indicates that the filtered output image is merely a coherent superposition of all possible images that are individually resulted from the integer OAM components. Besides, a real-time video of a rotating clover was recorded with the ideal SPP of 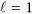. Our work may provide a deeper understanding of the spiral phase contrast imaging and may be anticipated to find potential application in the optical microscopy.

## Methods

### Experiment setup

Our optical system is sketched in Fig. 4, which can be effectively considered as a 4f system. Both Lens 1 and 2 have the same focal length of *f* = 50 cm. Two SLM (Hamamatsu, X10486-1) are employed in our experimental setup. The SLM1 and a color CCD camera are placed in the object plane and image plane of the 4f system, respectively, while the SPP filter displayed by SLM2 is accurately positioned in the Fourier plane. The input light is a vertically polarized Gaussian beam derived from a 5-mW, 633-nm He-Ne laser. After being collimated by a telescope, it is expanded and incident on SLM1. Each SLM is a reflective device consisting of an array of pixels (792 × 600), and each pixel imprints individually the incoming light with a phase modulation (0 ~ 2*π*) according to the 8-bit grayscale (0 ~ 255). Here we use SLM1 to display the pure phase object of a five-pointed star, whose profile is shown by inset (a) of Fig. 4. The interior and exterior areas of the star are completely out of phase, namely, they have a *π* phase difference. Besides, based on the polarization manipulation, we configure an optical isolator to improve the designability of an overall system, as it suppresses spurious interferences and undesired light routing^21^. This is realized by a polarizing beam splitter (PBS) combined with a quarter-wave plate (QWP@45°). The PBS transmits the horizontal polarization while reflects the vertical one such that the vertically polarized light beam after Lens 1 is first reflected by PBS and then directed to pass through QWP towards SLM2. The QWP in the double-pass configuration is equivalent to a half-wave plate (HWP@45°). Thus, after passing twice through QWP, the vertical polarization is converted into the horizontal one such that it can be subsequently transmitted by PBS. Thus the undesired back reflections is effectively avoided, yielding a higher detection efficiency. A trivial case is when SLM2 is not addressed to display any phase patterns then it acts as a mirror. The whole setup just performs the imaging function as a simple 4f system, and Eq. (6) can be simplified to, 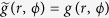. In contrast, if SLM2 is addressed to display the fractional SPP in the Fourier plane, then the Fourier spectrum of the pure phase object is modulated with an addition of a fractional spiral phase of exp(*iQφ*), as was described by Eq. (4).

## Additional Information

**How to cite this article**: Wang, J. *et al.* Gradual edge enhancement in spiral phase contrast imaging with fractional vortex filters. *Sci. Rep.* **5**, 15826; doi: 10.1038/srep15826 (2015).

## Supplementary Material

Supplementary video 1

Supplementary video 2
